# A Review of Novel Systemic Therapies and Brain Radiosurgery for Metastatic Melanoma

**DOI:** 10.4021/wjon506w

**Published:** 2012-07-05

**Authors:** Dexter A Overbay, Hakan Kaya, Wayne T Lamoreaux, Robert K Fairbanks, Alexander R Mackay, John J Demakas, Barton S Cook, Christopher M Lee

**Affiliations:** a Internal Medicine Residency Spokane, 101 W 8th Ave, Box 2555, Spokane WA, 99220-2555, USA; b Cancer Care Northwest, 910 W 5th Ave, Suite 102, Spokane, WA 99204, USA; c Gamma Knife of Spokane, 910 W 5th Ave, Suite 102, Spokane, WA 99204, USA; d Mackay and Meyer MDs, Spokane, WA 99204, USA; e Spokane Brain & Spine, Spokane, WA 99204, USA

**Keywords:** Hernia repair, Giant ventral hernia, Polypropylene mesh

## Abstract

Melanoma is a prevalent and particularly aggressive form of skin cancer. Although local disease can be treated well with excision, metastatic extracranial and intracranial disease can be very difficult to treat. Systemic therapies for melanoma have only recently led to statistically significant increases in overall survival with drugs such as Ipilimumab and Vemurafenib. Additionally, melanoma has been classically described as a relatively radio-resistant malignancy. Because of the potential radio-resistance, stereotactic radiosurgery has been widely utilized for intracranial brain metastases and clinical data has revealed excellent rates of local tumor control and tolerability with these highly focused radiation doses.

## Introduction

There are approximately 68,100 new cases of melanoma diagnosed in the United States each year and 8,700 deaths each year from this disease [1]. Metastatic melanoma has always been a challenging disease for the clinician due to poor treatment options and the aggressive nature of this cancer. While the traditional combination of biologic and cytotoxic agents have been shown to have an increase in relapse-free survival, the data has not shown a robust long-term increase in overall survival with metastatic disease [2-5].

Until recently, only three drugs had been approved by FDA in treatment of melanoma in more 35 years. These included approval of dacarbazine in 1975 for metastatic melanoma, approval of high dose interferon in 1995 for adjuvant therapy, and approval of high dose interleukin-2 in 1998 for treatment of metastatic melanoma. Following this 35 year period, in 2011 two novel drugs were approved by FDA in treatment of melanoma. Ipilimumab (anti-CTLA4 antibody) and Vemurafenib (V600 mutant BRAF inhibitor) were approved for systemic treatment of metastatic melanoma. Among the older drugs (Dacarbazine, high dose interferon and high dose IL-2), only high dose IL-2 was shown in the past to have a proven impact on overall survival [6-8].

Melanoma also has a predilection to metastasize to the brain and is the third most common cancer to do so [9]. While the reported rates of metastasis to the brain vary widely in the literature (20-40%), autopsy reports of patient with metastatic melanoma reveal the prevalence in some studies to be 75% [10-14]. It is inarguably significant and associated with a high rate of mortality. The median survival of patients with brain metastases who were treated with past therapies was on average two to four months [13, 15, 16]. Interestingly, only 40% of patients with brain metastases secondary to melanoma have a solitary mass [14]. The conventional treatment of choice for brain metastases has been surgical removal, however most brain metastases are not amenable to surgical removal with only approximately 19% of patients being candidates for surgical resection [14] Furthermore, the vast majority of mortality attributable to metastatic melanoma is due to brain metastases [14]. Thus, research into treatment advances such as stereotactic radiosurgery has led to improved intracranial local-control rates and survival for this challenging patient population. This emphasizes the need for novel systemic therapies and intracranial treatments with increased efficacy for metastatic melanoma.

## Discussion

The historic first line therapy for metastatic melanoma in patients with good organ function and performance status is Interleukin 2. Interleukin 2 is a cytokine that is an immune signaling agent that stimulates proliferation and differentiation of T cells. By giving high dose Interleukin 2 it is theorized that it stimulates an immune response against melanoma cells. High dose Interleukin 2 therapy has been shown to have better response rates than dacarbazine [6]. While Interleukin 2 has similar clinical tumor response rates as chemotherapy, it has shown to significantly improve survival while standard chemotherapy hasn’t [6]. Interleukin 2 therapy, however, can be significantly toxic. Side effects ranged from cardiac, pulmonary, hepatic, and infection. Hypotension was the most common side effect occurring in 64% of patients. Clinical reports revealed that 2.2% of patients died from adverse effects [6].

Ipilimumab was approved by the FDA for treatment of metastatic melanoma in March of 2011. It mechanistically binds to the CTLA-4 (cytotoxic T lymphocyte associated antigen 4) molecule on cytotoxic T lymphocytes. By binding to this molecule T lymphocyte activity is increased against melanoma cells. In the first published randomized clinical trail (RCT), ipilimumab, in combination with gp100 or alone, had significantly better survival than gp100 alone (10 months vs. 6.4 months) [17]. An additional RCT evaluated the difference between ipilimumab plus dacarbazine versus dacarbazine plus placebo and showed longer overall survival in the ipilimumab arm (11.2 months vs. 9.1 months) as well as increased 3-year survival rate (20.8% vs. 12.2%) [18].

Due to immune stimulation from ipilimumab, immune related toxicity and adverse effects were very common in patients receiving ipilimumab. In the first trial noted, 60% of patients had some side effect with the most common being diarrhea [17]. In this report, 10-15% of the patients had serious side effects requiring corticosteroid treatment and 14 deaths were attributable to the study drugs with 7 deaths associated with immune-related adverse effects [17]. The second trial had similar rates of adverse effects but no deaths attributable to the study drugs [18].

In metastatic melanoma patients, about 45% of their cancer expresses a specific mutation, V600, in an intracellular signaling kinase, BRAF [19]. Vemurafenib, a novel therapy for metastatic melanoma, has been shown to inhibit this mutated kinase. The first and only published RCT for vemurafenib compared this drug vs. dacarbazine in 675 patients. Vemurafenib improved overall survival and progression-free survival (six month survival 84% vs. 64%) with a RR of death being 0.37 and RR of death or progression being 0.26 (p < 0.001) [20]. Much like the ipilimumab however, there were significant reported side effects. In this series, 38% of patients needed dose adjustments due to adverse effects, with arthralgia being the most common. Significant photosensitivity was a common side effect as well and 18% of patients developed cutaneous squamous cell carcinoma or keratoacanthoma that require simple excision (Table 1) [20]. Due to the results of this study, vemurafenib and the lab test to detect the V600E mutation were approved by the FDA in 2011.

**Table 1:** Metastatic Melanoma Treatments and Effects

Treatment	Partial response (decrease in tumor size by at least 50%)	Complete response (no tumor detectable)	Overall Response	Median Overall Survival	Reference
Biochemotherapy vs. Chemotherapy	21% vs. 15%	7% vs. 3.5%	28% vs. 18.5%	No difference	2
Interleukin 2	10%	6%	16%	11.4 months	7
Ipilimumab vs. Chemotherapy	13.6% vs. 9.5%	1.6% vs. 0.8%	15.2% vs. 10.3%	11.2 months vs. 9.1 months	20
Vemurafenib vs. Chemotherapy	47% vs. 5%	1% vs. 0%	48% vs. 5%	At 6 months: 84% vs. 64%	22

Because the majority of systemic therapies have a challenge crossing the blood brain barrier, radiation therapy has been the mainstay of treatment for brain metastases from melanoma. Historically, whole brain radiation therapy (WBRT) has been used with or without surgery for the treatment of brain metastases. However recent clinical evidence shows that stereotactic radiosurgery (SRS) can have better survival rates and local control rates than WBRT alone [21]. SRS utilizes a very conformal, large dose of radiation to treat small volume areas containing metastases. It is usually a single treatment and is well tolerated. In Gamma Knife, for example, 201 beams are used to converge on the tumor to deposit a large volume of radiation but relatively sparing the surrounding normal tissue.

Multiple trials have been conducted that find SRS prolonging survival in patients with melanoma brain metastases from 2 to 4 months with conventional therapies to 7 to 8 months [22-25]. However, the addition of WBRT after SRS in some studies has been shown to decrease the rate of new brain metastases better than SRS alone. In one RCT, SRS plus WBRT had a 12 month recurrence rate of brain metastases of 46.8% while SRS alone had a recurrence rate of 76.4% [23]. SRS also has been shown to improve quality of life by decreasing headaches, visual disturbances, and seizures [26]. While melanoma is historically thought to be a relatively radio-resistant tumor type, the reported local control rates with SRS range from 75-97%. Furthermore, a large cost-effectiveness and cost-utility analysis done seems to favor radiosurgery as the most cost-effective treatment for any single-brain metastasis [27]. It should also be noted that ipilimumab and vemurafenib have also been shown in clinical trails to have activity against brain metastases by reducing tumor size [28-30].

To summarize, these novel treatments for metastatic melanoma led to improved survival rates and intracranial brain metastasis control, however the disadvantages must be addressed as well. Interleukin 2 can only be used for carefully selected patients have good organ reserve and performance status and has significant systemic toxicities [6]. Ipilimumab has serious autoimmune toxicity related to it and it takes months to see any clinical response, therefore making a poor choice in patients with large tumor burden. Vemurafenib has a response rate of 40-50% and a clinical effect can be seen in days to weeks, however the duration of response is only 5 - 6 months which is due to either another surface protein mutating creating an alternate pathway, or another protein in the BRAF pathway mutating rendering vemurafenib ineffective [19-20]. Although in general well tolerated, SRS has a small risk of familiar radiation adverse effects such as nausea, hair loss, tissue edema, or brain injury.

Oncologists remain optimistic about the future of metastatic melanoma research. There are currently multiple drugs in phase II and III trials that show promise in the treatment of this disease. Nilotinib, a cKIT inhibitor, has already been approved for use in CML, but is currently being studied for use in melanoma. Dabrafenib, another BRAF inhibitor similar to vemurafenib is under study as well. Allovectin-7 is a drug that incorporates a major histocompatability complex 1 into the tumor cells allowing the immune system to recognize the tumor cells and attack. These are just a few of the novel drugs that are in development and which show promise.

### Conclusion

Due to the addition of novel systemic therapies and advancements in brain radiosurgery for metastatic melanoma, survival and local control rates have been significantly increased. These new discoveries are stepping-stones for new research into more targeted therapies for this aggressive malignancy. Currently, research teams are looking into new mutations in melanoma cells to create novel targeted therapies that will be more efficacious and safer to use. In the meantime, patients with metastatic melanoma should be evaluated for the optimal available treatment and if available invited to participate in open clinical trials. Given the available data, interleukin 2, ipilimumab and verurafenib should be considered in each patient with metastatic melanoma. For patients with brain metastases, stereotactic radiosurgery with or without whole brain-radiation therapy should always be considered as well.
